# Triptycene Derivatives: From Their Synthesis to Their Unique Properties

**DOI:** 10.3390/molecules27010250

**Published:** 2021-12-31

**Authors:** Mateusz Woźny, Adam Mames, Tomasz Ratajczyk

**Affiliations:** 1 Institute of Organic Chemistry, Polish Academy of Sciences, Kasprzaka 44/52, 01-224 Warsaw, Poland; 2 Institute of Physical Chemistry, Polish Academy of Sciences, Kasprzaka 44/52, 01-224 Warsaw, Poland

**Keywords:** triptycene, synthesis, regioselective, sterically crowded, catalysis, molecular interactions, hydrogen bonds, nonbonding interactions, NMR

## Abstract

Since the first preparation of triptycene, great progress has been made with respect to its synthesis and the understanding of its properties. Interest in triptycene-based systems is intense; in recent years, advances in the synthetic methodology and properties of new triptycenes have been reported by researchers from various fields of science. Here, an account of these new developments is given and placed in reference to earlier pivotal works that underpin the field. First, we discuss new approaches to the synthesis of new triptycenes. Progress in the regioselective synthesis of sterically demanding systems is discussed. The application of triptycenes in catalysis is also presented. Next, progress in the understanding of the relations between triptycene structures and their properties is discussed. The unique properties of triptycenes in the liquid and solid states are elaborated. Unique interactions, which involve triptycene molecular scaffolds, are presented. Molecular interactions within a triptycene unit, as well as between triptycenes or triptycenes and other molecules, are also evaluated. In particular, the summary of the synthesis and useful features will be helpful to researchers who are using triptycenes as building blocks in the chemical and materials sciences.

## 1. Introduction

**Figure 1:**
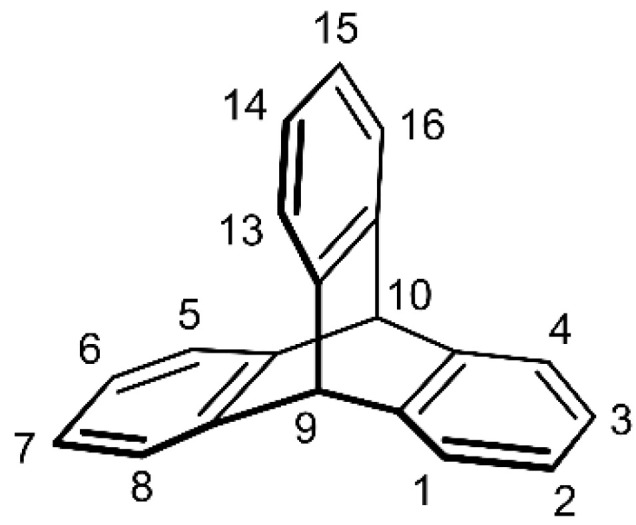
The chemical structure of the parent triptycene with a commonly used carbon atom numbering scheme based on the 9,10-dihydro-9,10[1′,2′]-benzenoanthracene nomenclature (CAS).

The parent triptycene (9,10-dihydro-9,10[1′,2′]-benzenoanthracene) is an eye-catching hydrocarbon with a paddlewheel or propeller-shaped molecule. The characteristic structure of triptycene is composed of three benzene rings joined by two sp^3^ carbon atoms in a *D*_3h_-symmetric structure with a barrelene core (Figure 1).

Triptycene was synthesized for the first time in 1942 by Bartlett et al., who also coined the name [1]. Fourteen years later, the rather laborious procedure became superseded by the more direct benzyne to anthracene cycloaddition route [2]. Since then, great progress has been made in the area of triptycenes synthesis, and recent developments are rich in examples of advanced structures, such as congested and structurally complex molecules [3,4,5,6,7].

At the same time, some efforts were made to provide convenient synthetic methods for the preparation of prefunctionalized triptycenes that could easily be used as robust synthons for function-focused derivatization. Such tools are needed because the interest in triptycene has gone beyond the area of purely synthetic curiosity and is now mainly driven by the useful properties of the molecule. The triptycene molecule is characterized by: (a) high reactivity due to the presence of several chemically distinguishable positions on aromatic rings and bridgehead atoms; (b) limited conformational freedom and well-defined spatial arrangement of substituents, making the triptycene a rigid and robust scaffold; (c) internal molecular free volume (IMFV) [8] due to the inability of triptycene-containing structures to pack efficiently, which introduces shape-persistent local cavities; as well as (d) the ability of aromatic faces to participate in π-interactions; and (e) the chirality of triptycene derivatives, which can arise from appropriate substitution patterns.

Due to these inherent characteristics, triptycene has become an exemplar of a multipurpose molecular scaffold [9,10,11,12,13,14,15,16,17,18,19,20,21,22]. Triptycenes were used as building blocks for mechanically interlocked molecules [23,24,25,26,27,28,29,30,31,32,33] and for self-assembly and molecular recognition in the field of supramolecular chemistry [7,34,35,36,37,38,39,40,41,42]; as achiral or chiral ligands for catalysis [43,44,45,46,47,48,49,50]; anti-cancer agents [51,52,53,54]; models of hindered rotation [55,56,57,58,59]; as well as in broadly defined materials sciences [60,61,62]; as structural units in various functional polymers and porous materials [63,64,65,66,67,68,69], such as molecular cages [18,70,71,72,73,74]; and metal–organic frameworks [75,76,77,78,79,80]; as well as in chemical sensors [81,82,83,84,85,86] and liquid crystals [87,88,89,90,91,92].

The interest in triptycene applications is growing steadily, and many new achievements have recently been reported. Therefore, we present a review of current progress in the field of triptycene chemistry that has not been described previously. We focus on two areas: the synthesis of triptycene derivatives and the understanding of the structure–property relationships that determine potential applications of triptycene in various fields of chemistry and material sciences. Such an account of triptycene chemistry has not been presented until now. This review can be helpful for researchers who wish to employ available synthetic scenarios for the preparation of triptycenes with the desired properties to be utilized in supramolecular chemistry, polymer science, and other fields.

## 2. Progress in Triptycene Synthesis and Derivatization

The efficient synthesis of triptycene derivatives is a challenge. Many synthetic routes have been developed and described in several excellent reviews [3,4,5,6,93]. Here, we would like to outline the major past achievements and discuss the results from recent works that have not yet been summarized.

### 2.1. General Synthesis of the Triptycene Unit

**Scheme 1:**
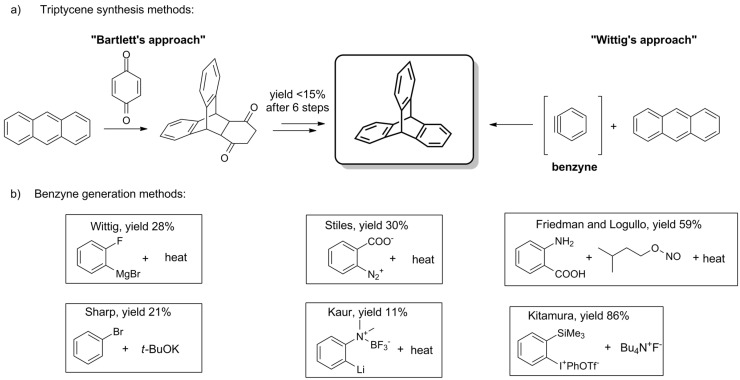
(**a**) The general synthetic pathways for the synthesis of triptycenes [1,2]. (**b**) Benzyne generation methods: Wittig [2], Stiles [95], Friedman and Logullo [96], Sharp [97], Kaur [98], Kitamura [99].

The parent triptycene was obtained for the first time in 1942 by Bartlett et al., who used anthracene and 1,4-benzoquinone as starting materials (Scheme 1a) [1]. The rationale for this rather laborious, low-yielding, multi-step synthesis was laid in the study of radicals that can be generated on Csp^3^ bridgehead carbon atoms of triptycene (positions 9 and 10, Figure 1) [1,5].

Although the procedure was later simplified by Craig both in terms of time and steps required [94], a true breakthrough came with the distinct discovery of a cycloaddition reaction between anthracene and a direct synthon of triptycene’s benzene ring—benzyne. In 1956, Wittig and Ludwig were the first to report such a reaction of benzyne (Scheme 1a), being a reactive intermediate generated in situ from 2-fluorophenylmagnesium bromide (Scheme 1b) [2]. A few years later, Stiles described the synthesis of triptycene using *o*-diazonium benzoate (Scheme 1b) [95].

In 1963, Friedman and Logullo found anthranilic acid to be a superior benzyne precursor (Scheme 1b) [96]. During the reaction, anthranilic acid undergoes seamless diazotization by amyl nitrite. The resulting benzenediazonium-2-carboxylate fragmentates giving N_2_, CO_2_, and benzyne, which in turn engages in Diels–Alder reaction with anthracene. The feasibility of the procedure manifests itself in the good yield (59%) and the availability of starting materials. To this day, various benzyne precursors have been presented (Scheme 1b), some of which allowed for the considerable improvement of the yield [97,98,99].

Variously substituted starting materials are within the scope of the Diels–Alder reaction between anthracenes and benzynes (“Wittig’s approach”) or 1,4-benzoquinones (“Bartlett’s approach”). Therefore, apart from the synthesis of the parent hydrocarbon itself, these reactions became the methods of choice for the synthesis of functionalized triptycenes. This type of situation is more out of necessity than choice, however, because the chemistry of the parent triptycene has its peculiar limitations resulting from regioselectivity issues and the relatively low reactivity of *ortho* (α) aromatic positions.

### 2.2. Synthesis of Ortho-Functionalized Triptycenes

Electrophilic aromatic substitution (S_E_Ar) reactions are disfavored at the *ortho* (α) positions respective to the bridgeheads. For example, nitration of the parent triptycene (HNO_3_/AcOH in Ac_2_O, 27–29 °C) yields products with the α to β substitution ratio of 1 to 40 [100]. This feature is a manifestation of the so-called “fused *ortho* effect” [101], according to which the reactivity toward electrophiles is largely diminished at the positions of the aryl ring that are *ortho* in respect to a fused strained ring [102]. In other words, a strained ring, such as triptycene’s bicyclo [2.2.2]octatriene, acts as a deactivating, *meta*/*para* directing group when fused with an arene.

Since nitrotriptycenes provide access to the corresponding amines and other derivatives, such as alcohols and halogenated compounds, some optimized synthetic procedures for nitration were developed with regioselectivity in mind. Even though there are six β positions in the parent triptycene, mononitration to 2-nitrotriptycene was achieved with as high yield as 58–64% [80,103]. Optimized dinitration protocols yield 2,6-dinitrotriptycene (39%) [104], or a mixture of 2,6-/2,7-dinitrotriptycenes (56%) [105], and trinitration gives 2,7,14- and 2,6,14-trinitrotriptycene (21% and 64%, respectively) [106]. The potentially explosive product of an exhaustive nitration, 2,3,6,7,14,15-hexanitrotriptycene, was obtained in 42% yield [105]. Other S_E_Ar reactions, such as acetylation [107,108] and formylation [108], also occur at β positions.

Because of the fused *ortho* effect, α-functionalized triptycenes are usually prepared from appropriate prefunctionalized substrates. 1,8,13-Functionalized triptycenes epitomize an example of such an α-substitution pattern. The significance of this family originates from the structure–property relationship. The symmetry and rigidity of the triptycene itself, combined with the advantage of the substituents being located on the same face of the scaffold (pointing in one direction), facilitate the juxtaposition of functional groups in proximity.

As tripodal molecules, 1,8,13-functionalized triptycenes recently found advanced application in surface modification—in 2015, Seiki et al. utilized the triptycene with three C12 side chains to self-assembly a remarkable organic thin film that was free of domain boundaries and structurally ordered up to the centimeter length scale [109]. The order and precise alignment of the molecular units of choice, which 1,8,13-functionalized triptycene allows for, are advantageous features for the promising field of high-performance organic thin-film materials [62,110,111,112]. The same 1,8,13 substitution pattern was used in models of molecular-level magnetic anisotropy [113], close nonbonded H–H contacts [114], and through-space Me–Ar interactions [115].

The first synthetic works on the 1,8,13 substitution pattern, in the 1970s and 1980s, concerned the synthesis of trichlorotriptycenes via Diels–Alder reaction of 1,8-disubstituted anthracenes with 3-substituted benzyne [113,116]. With the fused *ortho* effect in action, the synthetic route has remained unchanged ever since. Moreover, this is so despite the drawback—the formation of an *anti* (1,8,16-) isomer next to the desired *syn* product. The value of *syn*/*anti* ratio can be unfavorable (for example, ca. 1:3 to 1:4 for trichlorotriptycene [116,117]), and it depends on the substituents in a way that could be explained by the frontier orbital-controlled regioselectivity of cycloaddition [118].

**Scheme 2:**
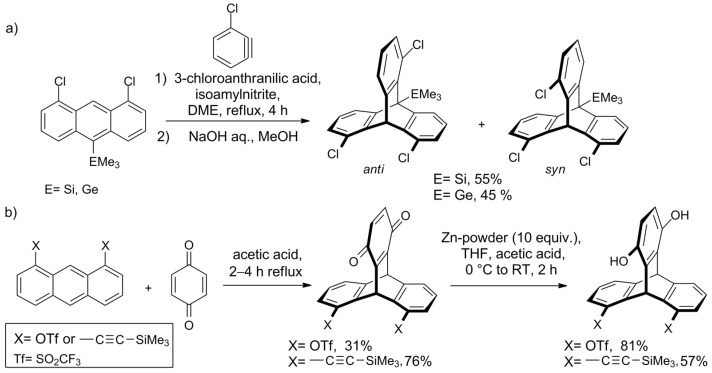
Studies on the regioselectivity of the synthesis of *ortho*-substituted triptycenes by the Mitzel group: (**a**) the electronic character of a substituent on C10 influences the *syn*/*anti* ratio of the product [119,120]; (**b**) triptycenes functionalized with triflate or (trimethylsilyl)ethynyl groups [120].

In 2010, the Mitzel group investigated the steric influence of the additional substituent at C-10 on the *syn/anti* ratio of 1,8,13/16-trichlorotriptycenes [117] and found that *anti*-isomer is generally favored. Unexpectedly, this was true for large substituents too, so the Mitzel group changed their focus from steric to electronic effects. Recently, they have investigated the cycloaddition of 3-chlorobenzyne to three 1,8-dichloroanthracenes equipped with CMe_3_, SiMe_3_, or GeMe_3_ group in position 10 [119]. It was observed that the electronic properties of the substituent influence regioselectivity—with electropositive SiMe_3_ and GeMe_3_, the *syn* triptycene formed predominantly, whereas for CMe_3_ only an *anti*-isomer was observed (Scheme 2a). Quantum chemistry calculations of the reaction pathway revealed that dispersion forces are one of the factors responsible for the observed effects.

Another approach aimed at regioselective synthesis of triptycene derivatives was presented by the group in 2018—Shwartzen et al. used cycloaddition of 1,4-benzoquinone to substituted anthracenes for the preparation of triptycenes functionalized with triflate or C≡C-SiMe_3_ groups (Scheme 2b)[120].

For the intermediate with two sterically demanding SiMe_3_ groups, a distortion of the molecular framework was observed. For that reason, attempts to obtain the 1,8,13-trisubstituted derivative by introduction of the third C≡C-SiMe_3_ were unsuccessful.

On the other hand, since the regioisomeric course of the Diels–Alder reaction depends on the nature of substituents in prefunctionalized substrates, approaches departing from chloroderivatives are also being explored. In their work reporting the synthesis of a porous molecular cube, Elbert at al. presented a path towards 1,8,13-trihydroxytriptycene [118], starting with the Diels–Alder reaction of 1,8-dimethoxyanthracene and 3-methoxybenzyne. The latter was generated in situ by a mild, fluoride-induced 1,2-elimination [121] of the corresponding trimethylsilyl triflate precursor [122]. A poorly soluble 2:1 mixture of *syn*- and *anti*-trimethoxytriptycenes was collected, and the target 1,8,13-trihydroxytriptycene was obtained after ether cleavage, esterification with capronyl chloride, crystallization of the desirable regioisomer, and hydrolysis of the pure 1,8,13-triester. The same method was used in the above-mentioned work of Seiki [109], who reported that *syn*-trihydroxytriptycene can be separated from the *anti*-isomer by recrystallization from DMF at −35 °C.

**Scheme 3:**
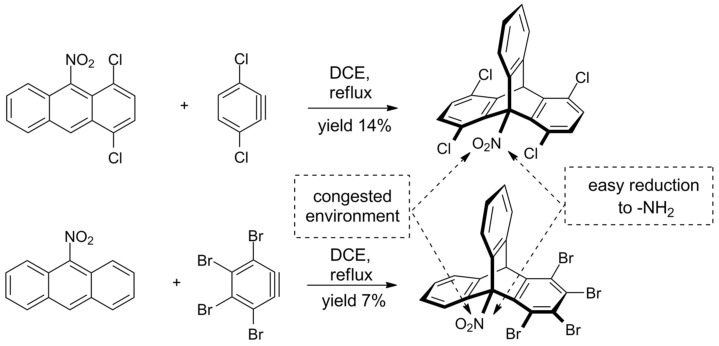
Synthesis of new *ortho*-functionalized triptycenes from deactivated, electron-deficient nitroanthracenes [123].

The challenge of sterically crowded triptycene synthesis was also taken by Szupiluk, who presented a route towards 9-nitro- and 9-aminotriptycenes (Scheme 3) [123]. The procedure comes down to cycloaddition of benzynes to 9-nitroanthracenes, followed by reduction of the nitro group. The reaction of electron-deficient anthracenes was not regioselective and accompanied by the formation of considerable amounts of side products. For example, 1,2,3,4-tetrabromo-9-nitrotriptycene was obtained in 7% yield; two other products were identified. On the other hand, it was demonstrated that the final reduction of nitro group on C9 in triptycenes proceeds smoothly and in a good yield, giving products equipped with the synthetically useful ammonium group.

**Scheme 4:**
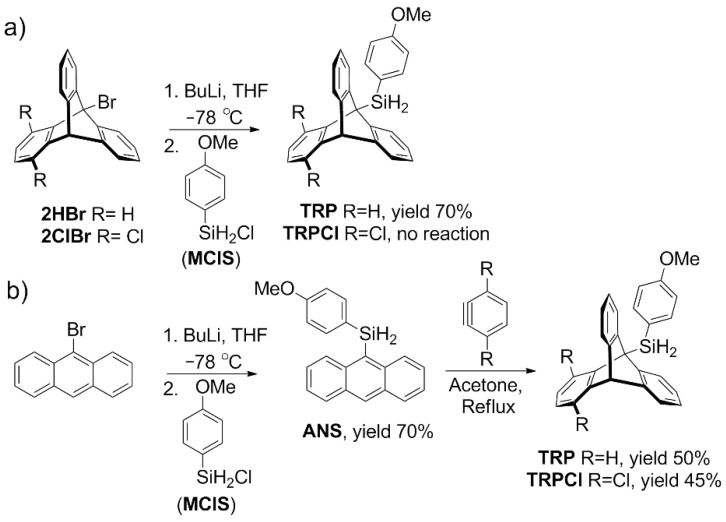
The two routes towards a sterically congested silyl triptycene derivative, via: (**a**) carbanion of the triptycene scaffold or (**b**) cycloaddition of prefunctionalized anthracene [124]. Copyright Royal Society of Chemistry.

On the one hand, the presence of steric congestion in the target triptycenes may cause some problems during its synthesis, but on the other hand it imparts unique properties. This was exactly the case with 1,4-dichloro-9-(*p*-methoxyphenyl)silyltriptycene (TRPCl), for which two synthetic routes were investigated in 2019 (Scheme 4) [124].

The first one involved the generation of a carbanion (Scheme 4a) on the Csp^3^ bridgehead, which was expected to react further with *p*-MeO-C_6_H_4_-SiH_2_Cl. However, no desired product was observed. In contrast to this, a less sterically congested 9-(*p*-methoxyphenyl)silyltriptycene (TRP) was prepared by both carbanion as well as via the cycloaddition route. The cycloaddition route involves less sterically congested intermediates (Scheme 4b). Therefore, sterically congested TRPCl can be synthesized via this route.

**Figure 2:**
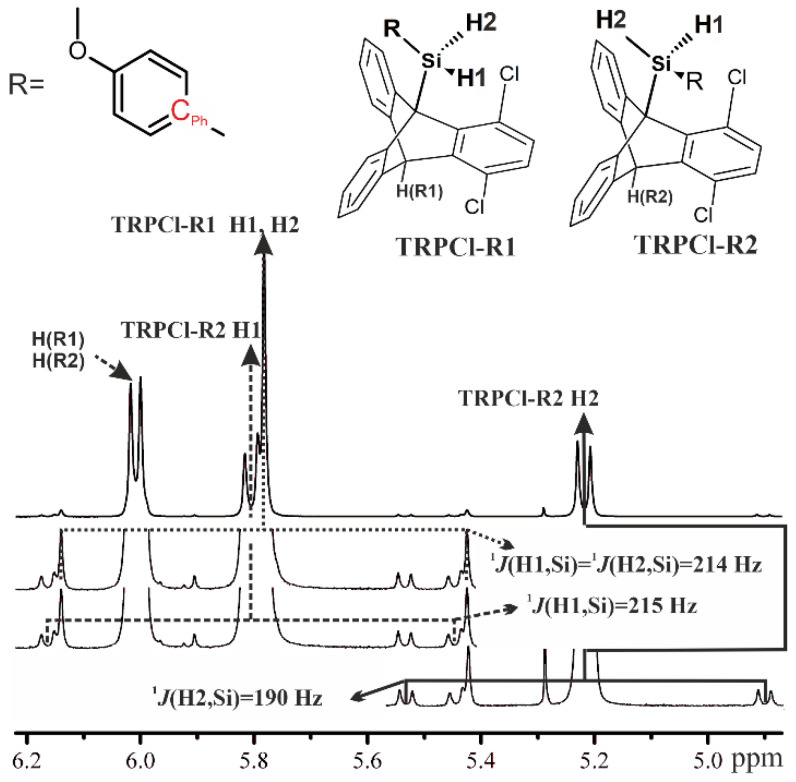
^1^ H NMR spectra for chloroform-*d* solutions of TRPCl, which exists in the form of two rotamers, TRPCl-R1 and TRPCl-R2 [124]. Copyright Royal Society of Chemistry.

The steric congestion in TRPCl manifests itself in the liquid state ^1^H NMR spectra (Figure 2), which reveals that TRPCl exists in the form of two rotamers in a ratio of about 1:2, as one of them exists in the form of two enantiomers.

The presence of the rotamers can be directly deduced from the ^1^H NMR spectra. In one of the rotamers, two silyl protons form the A2 spin system, whereas in the case of the second rotamer, the silyl protons form the AB spin system. In the case of the A2 spin system, a single resonance is observed at 5.79 ppm, while for the AB spin system two doublets are present in the spectrum, at 5.23 and 2.83 ppm, respectively (Figure 2).

Further detailed NMR experiments as well as quantum chemistry calculations disclosed a unique interaction between the electron-rich chloride in the peri position and the silyl group [125]. This interaction was possible to observe via the analysis of spin–spin coupling constants within the silyl group (Table 1). For example, substantial differences exist between the *anti*- and *syn*-located Si−C_Ph_ in TRPCl-R1 and TRPCl-R2, respectively. In the solid state, only a more stable rotamer is present, as confirmed by powder X-ray analysis.

Although the cycloaddition approach involving anthracene remains the most important synthetic route towards triptycenes, some fundamentally new syntheses are under development. For example, in 2007, Taylor et al. reported that using readily available anthraquinones and alkynes as starting materials, 9,10-dihydroxytriptycenes can be obtained with great yields in two to three steps [126]. The key transformation was a rhodium-catalyzed [2 + 2 + 2] cycloaddition.

**Scheme 5:**
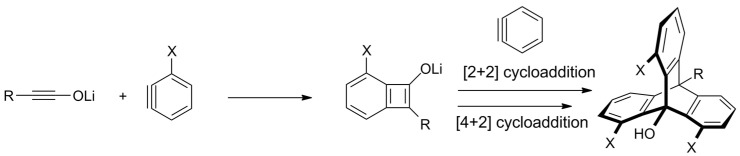
A new approach towards substituted triptycenes—a regioselective synthesis from in situ generated ynolates and benzynes (X = H or OMe for R = Me, *i*-Pr, *n*-Bu, or Ph; X = *t*-Bu and R = H; X = *n*-Hex and R = OMe) [128].

An excellent example of a new approach towards the synthesis of substituted triptycenes comes from Shindo’s group and their research on ynolates [127]—Umezu et al. reported that triptycenes can be obtained from benzynes and ynolates of the formula R-C≡C-OLi (Scheme 5) [128]. The reaction is carried out in one pot. Both benzyne and ynolate are generated in situ from fluorobenzene and α,α-dibromoester, respectively. Resulting triptycenes are substituted at the bridgehead atoms with ynolate-originating groups OH and R (Me, *i*-Pr, *n*-Bu, *t*-Bu, Ph, or Hex).

The uniqueness of this reaction outcome asks for a closer look. First, C≡C group of the ynolate is converted to triptycene’s bridgehead carbons. It means that in the reaction, a complete dismantlement of the carbon–carbon triple bond occurs, which, as the authors put it, represents “a conceptually unique application of alkynes in synthesis”. Second, in contrast with common syntheses involving anthracene, the product forms in a sequence of three cycloadditions: the initial one between benzyne and ynolate, and the other two between benzyne and consecutive intermediates. Therefore, all three benzene rings in the obtained triptycene originate from the benzyne.

The scope of the reaction was investigated by treating various ynolates with an excess of benzyne or 3-methoxybenzyne. Target triptycenes were obtained in yields ranging from 27% to 69%. Interestingly, experiments with 3-methoxybenzyne revealed high regioselectivity of the reaction. It proceeds in a head-to-head-to-head manner, so that all methoxy groups land on the same side of the obtained triptycene molecule (analogous regioselectivity was observed for disubstituted benzyne derivatives). The quantum chemical calculations showed that the two main driving forces behind the regioselectivity were chelation and negative hyperconjugation.

**Scheme 6:**
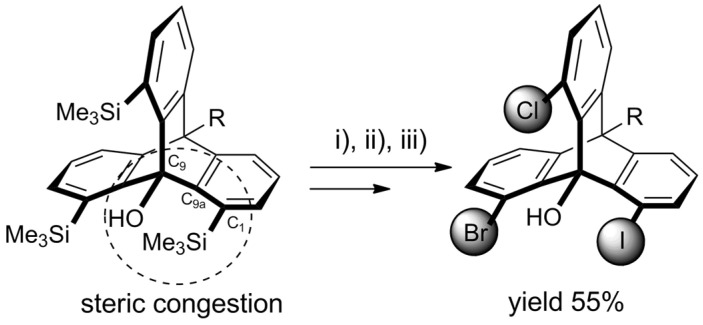
Trisilyltriptycene (obtained according to Scheme 5 for R = Me and X = SiMe_3_) as a starting material for heterosubstituted triptycenes. An exemplary 1-bromo-8-chloro-13-iodo product was obtained with 55% overall yield in a chromatography-free procedure involving selective halogenations with (i) NCS (1.2 eq. in DMF, 60 °C, 2.5 h); (ii) NBS (2.0 eq. in DMF, 60 °C, 24 h); (iii) ICI (2.0 eq. in DCM, rt, 1 h) [129].

Recently, Yoshinaga et al. published a follow-up article reporting some new developments in ynolate/benzyne chemistry [129]. The group presented the synthesis of 1,8,13-trisilylated triptycenes obtained from 3-silylbenzynes via the above-described triple cycloaddition route (Scheme 6). Consequently, a series of selectively substituted triptycenes was presented. This time, however, there were three bulky silyl groups in an “all-*syn*” relation, and the resulting steric congestion distorted the molecule, as evidenced by X-ray crystallography. For example, in triptycene 1,8,13-trisubstituted with SiMe_3_ groups, the dihedral angle of Si-C1-C9a-C9 was 21.7°, compared with <5° for triptycene with no such substituents. The authors carried out a substitution of SiMe_3_ groups with chlorine and bromine (using *N*-halosuccinimides) and found it to occur with high selectivity—depending on the stoichiometry, mono-, di-, or trihalogenated triptycenes were obtained in excellent yields. Reactions proceed through a nonstatistical mixture of products because the magnitude of strain released with each consecutive SiMe_3_ replacement decreases.

Yoshinaga’s method is of practical use because triptycenes 1,8,13-trisubstituted with various silyl/halogen combinations can be obtained and further derivatized accordingly. This method facilitates the synthesis of various triptycene derivatives that would otherwise be difficult to obtain. In practice, the feature was utilized in efficient preparation of a triptycene derivative with 1-bromo-8-chloro-13-iodo substitution pattern (Scheme 6).

### 2.3. Functionalization of the Bridgehead Positions of the Triptycene Unit

In the chemistry of triptycene, the desired substitution pattern dictates the synthetic strategy. Low reactivity of triptycene’s aromatic *ortho* positions contrasts with the well-known activated character of the analogous position in simple alkyl-substituted arenes. In the same vein, while a benzylic position is generally reactive, the bridgehead C-H groups in triptycene are relatively inert to many reactions. Therefore, both *ortho* and 9,10-functionalized triptycenes are usually obtained from already functionalized precursors.

Halogen groups incorporated in positions 9 and 10 of triptycene react with organometallic compounds (carbanionic character of the bridging carbon [130]), but they do not undergo typical substitution reactions. The S_N_1 mechanism is disfavored because the required carbocation would be non-planar and not stabilized by conjugation with aromatic rings. On the other hand, S_N_2 reactions are out of question by virtue of the presence of two bridgehead carbon atoms that shield themselves from the attack of a nucleophile.

**Scheme 7:**
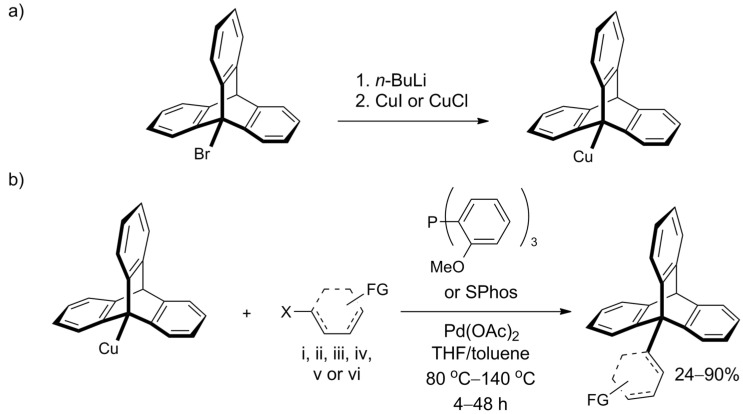
An efficient coupling at position 9 of the triptycene, reported by Oi et al. [131]: (**a**) The preparation of the Cu derivative of triptycene (**b**) The coupling partner is composed of X = I or Br and an aromatic system (represented by a dashed structure) such as: (i) phenyl; (ii) benzene ring with a functional group (FG): *o*-CO_2_Me, *p*-CO_2_Me, *p*-CF_3_, *p*-CN, *p*-Ac, *o*- or *p*-OMe; (iii) thiophenyl substituted with FG=Ac or CO_2_Et; (iv) pyridinyl; (v) quinolinyl; or (vi) ferrocene.

However, Oi et al. recently disclosed a Pd-catalyzed cross-coupling protocol that offers efficient functionalization of the bridgehead carbon atom in 9-bromotriptycene through C9-C bond formation (Scheme 7a) [131]. Various iodo- and bromoaromatic compounds were successfully tested as coupling partners, leading to a library of C9-Csp^2^ coupled products, which were obtained in good yields despite inherent steric hindrances. Illustrative C9-Csp coupling with (iodoethynyl)benzene and C9-Csp^3^ coupling with methyl bromoacetate were also successful. The procedure constitutes a highly attractive way of triptycene extension—its scope seems wide, the conditions are mild, and isolated yields were usually between 60% and 90%. Moreover, the required reagents—Pd(OAc)_2_ and tris(*o*-methoxyphenyl)phosphine (SPhos ligand)—are commercially available. 9-Triptycenylcopper, an active participant in the coupling reaction, forms in situ from 9-bromotriptycene treated with *n*-butyllithium followed by copper(I) halide (Scheme 7b). Additionally, the authors performed screening of other 9-metalated triptycene derivatives and found that 9-triptycenylboronate ester and 9-triptycenylzinc were not reactive under the tested conditions. In addition to triptycene, the scope of the work included C-C bond formation with other hindered structures—namely, adamantyl and mesityl.

In the case of the above route, the role of the metal type was explained with DFT calculations and Natural Bond Orbital (NBO) analysis—in the transmetalation step for an arylpalladium complex and 9-triptycenylboronate high energy transition states and high activation energy occur [131]. Although the corresponding values for 9-triptycenylzinc were more favorable, the transmetalated product appeared unstable thermodynamically, rendering the transmetalation improbable. None of such issues occur in Cu-metalated pathways. High-energy d orbitals, vacant coordination sites of copper, and Cu(I)–Pd(II) interaction turned out to be the key to the efficiency of copper-mediated cross-coupling.

### 2.4. Synthesis of Chiral Triptycenes

In the context of the structure–property relationship, another advantageous feature of the triptycene scaffold is its prochirality [132]. Indeed, many triptycenes with appropriate substitution patterns are optically active. The bridgehead carbon atoms can be seen as prochirality centers that can become asymmetric upon differentiation of their four substituents. For example, both 1,5- and 2,6-disubstituted triptycenes can, in principle, exist as two non-superimposable mirror images. The same holds true for 1,8- and 2,7-disubstituted triptycenes, provided that the substituents are different. Owing to the rigidity of the triptycene scaffold, the inherent chirality of such derivatives is permanent—there is no “risk” of racemization or inversion of configuration during chemical transformations. In 2020, an interesting review on chiral triptycenes in supramolecular and materials chemistry was presented by Preda et al. [7]. It is envisioned that chiral triptycenes will find applications in the growing field of circularly polarized luminescence (CPL) as CPL-active materials [133,134,135,136,137,138].

Sonoda reported enantiopure triptycenes obtained by recrystallisation of diastereomeric salts as early as in the 1960 s [139]. Recently, Ikai described a triptycene-based stationary phase for chiral HPLC [140]. The phase was effective in the resolution of axially chiral biaryls. The synthetic pathway began with a valuable racemic precursor, 2,6-dinitrotriptycene, obtained according to one of the above cited, optimized procedures [104].

**Scheme 8:**
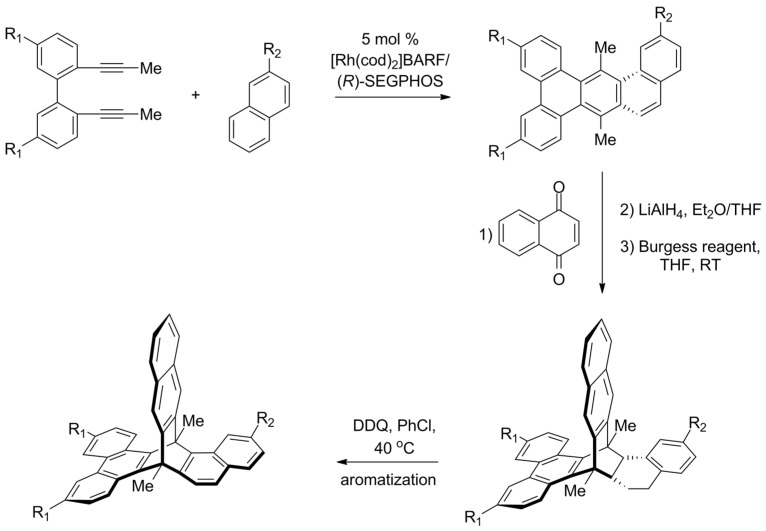
Aida’s enantioselective synthesis of π-extended triptycene precursors, in which all three “paddles” are different (R_1_ = H or CF_3_, R_2_ = H or OMe) [142]. The last aromatization step was successful only for CF_3_/OMe combination of substituents.

In 2015, the first enantioselective synthesis of chiral triptycene was reported by Shibata [141]. Another one was recently presented by Aida [142], who proposed a strategy that starts from the preparation of chiral multicyclic cyclohexadienes. A set of such compounds was obtained by a highly enantioselective [2 + 2 + 2] cycloaddition between 2,2′-di(prop-1-yn-1-yl)-1,1′-biphenyls and 1,2-dihydronaphthalenes (Scheme 8). The reaction was catalyzed by a complex of rhodium(I) with a chiral (*R*)-SEGPHOS ligand, and the resulting cyclohexadienes were obtained with high enantiomeric excess. Subsequent Diels–Alder reaction with 1,4-naphthoquinone followed by reductive aromatization gave triptycene precursors. The final oxidative aromatization step was expected to convert the precursors to target triptycenes, but it turned out successful only in one of the cases. Nevertheless, a π-extended chiral triptycene with all three aromatic rings being different was obtained (87% ee).

To explain differences in the behavior of precursors, the authors investigated mechanistic aspects of the carbocation-involving oxidative dehydrogenation. It was determined that high strain causes an undesired carbocation rearrangement, which could be suppressed by the use of the electron-rich alkene and electron-deficient diyne.

### 2.5. Triptycenes in Catalysis

Triptycenes can be substituted with phosphorous, both on aromatic rings, as well as on bridgehead atoms. For example, over 20 years ago, Ramakrishnan presented 9-phosphinotriptycene [143] as a model for the study of the restricted rotation in the solid state (the compound was obtained from 9-lithiotriptycene and PCl_3_, followed by LiAlH_4_). Bearing in mind the rigid structure of the triptycene scaffold and the synthetic availability of its phoshine derivatives, an important branch of triptycene-related research emerged in recent years—the chemistry of triptycene-based phosphine ligands for catalysis. The area was pioneered by Gelman et al., both in the context of ligand synthesis, as well as their catalytic utilization [43,44,45,46,144,145,146,147,148].

**Scheme 9:**
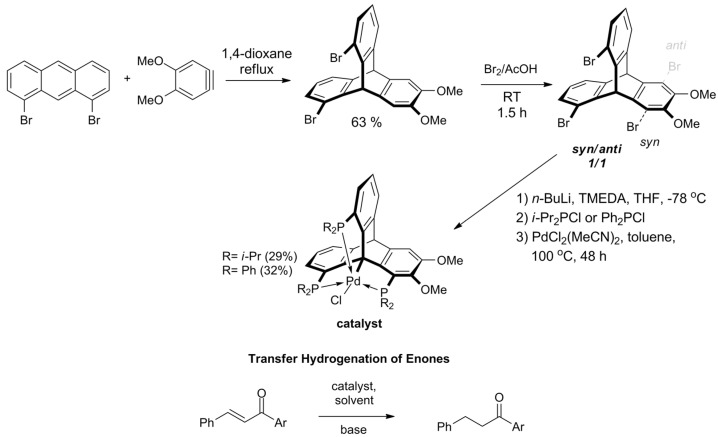
The first applicable synthesis of 1,8,13-tribromotriptycene. Phosphination and bridgehead carbon atom metalation gives triptycene-based complexes of Pd(II), which act as catalysts for chemoselective transfer hydrogenation of α,β-unsaturated ketones (R = *i*-Pr; Ar = Ph, 4-MeO-phenyl, 1-naphthyl, 2-furyl) [149].

In 2018, Kisets et al. presented a successful synthetic route towards 1,8,13-halogenated triptycenes and corresponding triphosphine ligands [149]. The method facilitates efficient synthesis of 1,8,13-tribromotriptycene derivative in two steps, starting with the Diels–Alder reaction of 1,8-dibromoanthracene towards 1,13-dibromo-6,7-dimethoxytriptycene (Scheme 9). Then, the intermediate was brominated giving a 1:1 mixture of *syn*- and *anti*-tribromotriptycenes that were separated by crystallization. Phosphination of the isolated *syn*-isomer yielded a pincer-like ligand with a rigidly arranged triphosphine coordination sphere. The reaction with PdCl_2_ resulted in carbometalation on Csp^3^-bridgehead atom. As evidenced by X-ray diffraction analysis, the geometry around the metallic center of this triptycene-based complex of Pd(II) was almost perfectly trigonal bipyramidal. It turned out that the compound is catalytically active during transfer hydrogenation of α,β-unsaturated ketones. The reaction is chemoselective owing to the singular steric environment around the catalytic site.

**Scheme 10:**
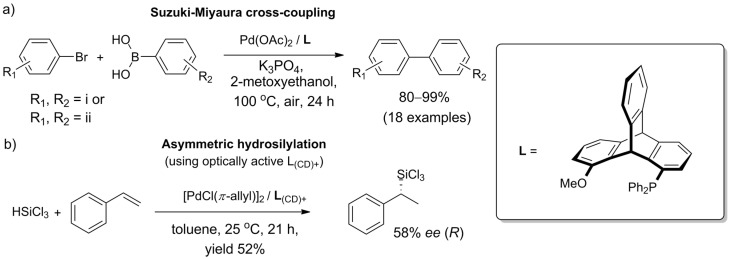
Catalytic utility of triptycene-based monophosphine ligand obtained by Leung et al. [150]: (**a**) in Suzuki–Miyaura cross-coupling with racemic ligand, the scope was successfully tested with the following substituents: (i) R^1^ = *o*-Me, *o*-OMe, *m*-OMe, *m*-NH_2_, *p*-Me, *p*-OMe, *p*-COMe, *p*-CHO, *p*-NO_2_, or *p*-CF_3_, provided that R^2^ = H; and (ii) all combinations of R^1^ = *p*-NO_2_, *p*-COMe, or *p*-OMe with R^2^ = *p*-CF_3_, *m*,*p*-(OMe)_2_, or *o*-Me; (**b**) in asymmetric hydrosilylation of styrene, chiral ligand facilitated the formation of chiral product (58% ee, as determined for the corresponding alcohol obtained via Fleming–Tamao oxidation.

The methods for the synthesis of catalysts, combined with the chiral nature of many derivatives, open the prospect for the deployment of triptycenes in asymmetric synthesis. The first case of enantioinduction due to a chiral monophosphinotriptycene-containing catalyst was reported in 2018. Leung et al. [150] noted that while bisphosphine ligands based on 1,8-disubstituted triptycenes are well researched, monophosphinotriptycenes are no less interesting—in general, monodentate ligands are highly active in metal-catalyzed reactions. The authors efficiently prepared an exemplary ligand, 1-methoxy-8-(diphenylphosphino)triptycene, in five steps starting from 1,8-dihydroxytriptycene. By design, the desymmetrization of the molecule occurred upon P(O)Ph_2_ group introduction, and the enantiomers were separated by chiral chromatography of the penultimate intermediate. Next, the catalytic activity of a monophosphinotriptycene scaffold was demonstrated for the first time in tests against Pd-catalyzed Suzuki–Miyaura cross-coupling. Although the racemic ligand allowed for biaryls to be obtained in excellent yields (>90% in most cases, Pd(II) at 0.001 mol%) (Scheme 10a), no enantiomeric excess was observed in tests utilizing an optically active triptycene for the coupling of 2-methylphenylboronic acid with 1-bromo-2-methylnaphthalene or 1-bromo-2-methoxynaphthalene. However, in Pd-catalyzed asymmetric hydrosilylation of styrene, optically active triptycene-based monophosphine ligand succeeded at induction of chirality—an expected product was obtained with a moderate enantiomeric excess (Scheme 10b). Bearing in mind that the stereochemistry of the catalyst’s molecular framework was relatively simple, the utilization of such a system in the context of asymmetric synthesis seems promising.

**Scheme 11:**
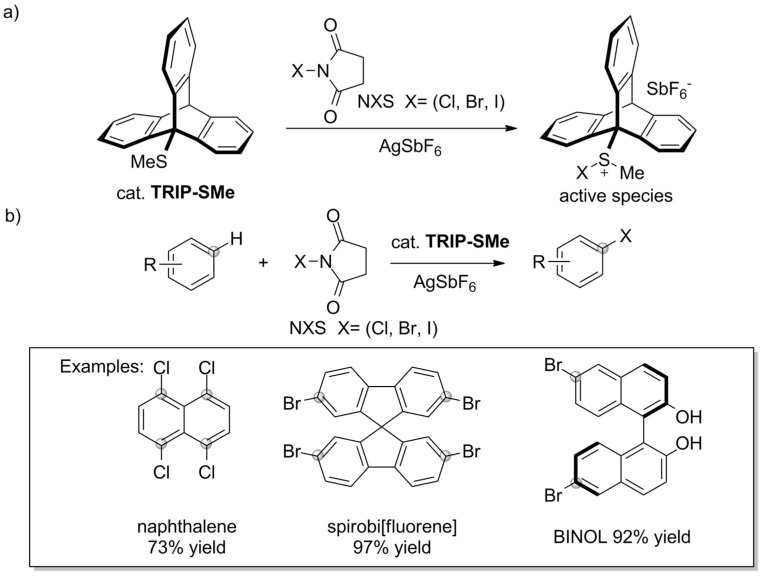
(**a**) Methylsulfanyltriptycene/AgSbF_6_ catalyst system reported by Nishii et al. [151] and (**b**) the mild halogenation of aromatic compounds by *N*-halosuccinimides.

The catalytic uses of triptycene go beyond the sphere of phosphine ligands. Some interesting results were recently presented by Nishii et al. [151]. The authors reported that 9-methylsulfanyltriptycene (TRIP-SMe) catalyzes the halogenation of aromatic compounds, including unactivated ones, by *N*-halosuccinimides (NXS) (Scheme 11a). The approach offers a route towards haloarenes in which harsh reaction conditions and/or classical electrophilic halogenation using bromine or chlorine can be avoided. TRIP-SMe catalyst was easily prepared by treating 9-bromotriptycene with *n*-BuLi and dimethyldisulfide, followed by two-step purification by chromatography and recrystallization (84% yield). The synthetic procedure, which allowed for successful halogenation of various aromatic compounds under mild reaction conditions, combines the aromatic substrate, NXS, TRIP-SMe catalyst, and AgSbF_6_ activator (Scheme 11b). According to the proposed mechanism, TRIP-SMe captures halogen from NXS to become a halonium adduct, TRIP-S(Me)X^+^ (Scheme 11a). Due to the presence of triptycenyl group, a significant charge separation occurs making the halogen atom highly positive and reactive in electrophilic aromatic substitution. Kinetic studies, X-ray data, and NPA calculations allowed the authors to conclude that the triptycenyl sulfide moiety plays a central role in the superior catalytic activity of TRIP-SMe; it was suggested that the high electrophilicity of the halogen atom could originate from the spatial organization of C-S bond and π-system of triptycene. The acknowledgement of the role played by triptycene was corroborated by experimental data showing the catalytic ineffectiveness of analogous systems devoid of triptycenyl groups.

## 3. Triptycenes: From the Inter- and Intramolecular Interactions to the Properties

Triptycene derivatives are well recognized as convenient models for the investigation and observation of different phenomena, including, for example, subtle quantum effects, optical and photophysical effects, and molecular interactions [55,152,153,154,155,156]. There are no review articles devoted directly to the topic; however, much information on intra- or intermolecular interactions of triptycene molecules or their crystals are covered, in a more or less scattered form, by reviews concerned with the synthesis of triptycenes. A large part of the review by Szymanski is concerned with triptycene derivatives as a model for the investigation of the Damped Quantum Rotation (DQR) theory [157]. Therefore, some of the most interesting examples of triptycene probes are briefly summarized herein to serve as an asset for the researcher who wants to have a general outlook on the issue.

### 3.1. Triptycenes and Molecular Interactions

Here, we focus on triptycenes as molecular probes for the investigation of molecular interactions and photophysical phenomena. Molecular interactions can be investigated by various methods such as Nuclear Magnetic Resonance Spectroscopy (NMR). Triptycene’s positions 9 and 2 are of central importance for the investigation of their mutual interaction because NMR studies are greatly facilitated by the dynamic properties of the sp^3^-bridgehead substituent. More precisely, the dynamics of this substituent can be frozen on the NMR time scale, and afterwards, details of the mutual interaction become visible. However, detailed studies of molecular interactions in triptycenes are not limited to 2,9-functionalized triptycenes, and other models were also used.

**Figure 3:**
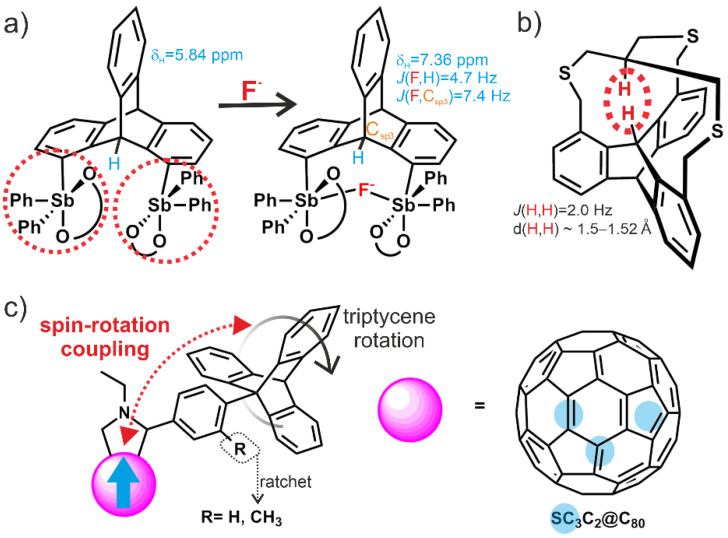
(**a**) A weak interaction between Csp^3^-H and F^−^ is confirmed by the spin–spin coupling constants [158]. (**b**) The congested structure of 1,8,13-tris(mercaptomethyl)triptycene and the interaction between two closely located protons (**c**) The spin-rotation coupling within the triptycene–fullerene-based system [159].

For example, a weak C–H∙∙∙F interaction was identified in the triptycene-based distiborane (Figure 3a) [158]. This triptycene derivative is a bidentate Lewis acid that can bind fluoride anions between two Sb units. Direct interaction between the coordinated fluoride anion and the hydrogen atom at the sp^3^-bridgehead position was detected via NMR spectra, in which it was visible that the hydrogen bond interaction with fluoride affects the methine proton resonance and shifts it downfield from *δ* = 5.84 for bare triptycene to 7.36 ppm in the case of the complex. This hydrogen bond is responsible for the stabilization of the anionic complex. Interestingly, the proton resonance appears as a doublet with ^1^*J*_HF_ = 4.9 Hz, and the coupling between fluoride and the bridgehead carbon is also coupled with *J*_CF_ = 7.4 Hz.

Another interesting example of triptycene in which a unique interaction is present was discussed by Pascal et al. [114]. In this work, the 1,8,13-tris(mercaptomethyl)triptycene was closed from the top by tris(bromomethyl)methane, yielding triptycene-based *in*,*in*-cyclophanes (Figure 3b). In this molecule, two protons, one bonded to the triptycenic bridgehead carbon atom and the other residing on the ternary carbon of the mercaptomethyl unit, are pointed towards each other. Based on X-ray data and DFT quantum chemistry calculations, the distance between these two protons is small, amounting to approximately 1.5–1.53 Å. As a result of this hydrogen–hydrogen non-bonded contact, NMR indicated a spin–spin coupling constant equaling 2.0 Hz between two in-hydrogen atoms, which is a large value as for the coupling through space.

The sp^3^-bridgehead position can also be employed for the integration of the triptycene skeleton with a matallofullerene (Figure 3c) [159]. Accordingly, Meng prepared 4-triptycenebenzaldehyde and 3-methyl-4-triptycenebenzaldehyde (Figure 3c). In the latter derivative, the methyl group works as a ratchet, which introduces the steric hindrance, and therefore restricts the rotation of the benzophenone ring around the C_3_ triptycene axis. These two derivatives were mounted on a paramagnetic metallofullerene Sc_3_C_2_@C80 via a covalent bond, which created two binary systems that are excellent models for the investigation of how the spin-rotor coupling is influenced by the triptycene unit rotation speed. More precisely, the rotation of the triptycene unit is fast on the NMR timescale in the TRP-Sc_3_C_2_@C80, and it is restricted in TRPMe-Sc_3_C_2_@C80. Furthermore, the dynamics of the triptycene moiety change with temperature, which is evidenced by the NMR spectra from which the constant rate can be evaluated. Regarding all of this, it turned out that the rotations of mounted triptycene units influence the spin–metal hyperfine couplings as well as the electron spin relaxation processes, which is clearly visible from EPR experiments.

**Figure 4:**
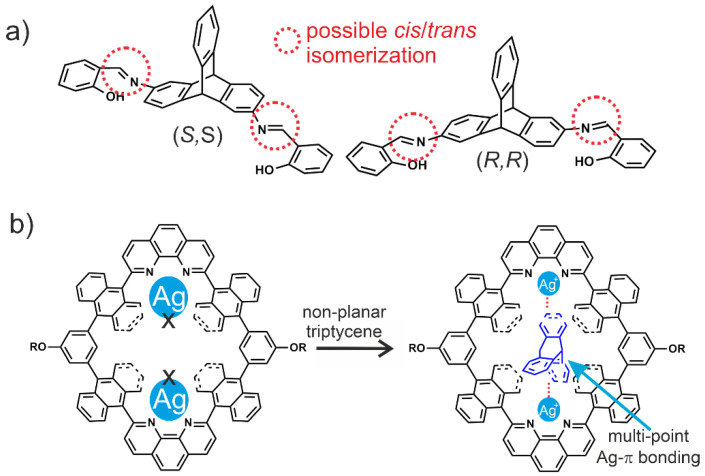
(**a**) The triptycene derivative with imine C=N bonds, which can induce isomerism [160]. (**b**) A host–guest system in which a multipoint Ag–π bonding facilitates recognition of a triptycene molecule by a Ag(I)-containing macrocyclic host [161].

Interesting molecular architecture in the form of trinuclear circular helicate was presented according to the example of two triptycene enantiomers, which were products of the condensation of 2,6-diaminotriptycene and 2-hydroxybenzaldehyde (Figure 4a) [160]. These enantiomers contain imine C=N bonds giving rise to conformational isomerism. Both enantiomers are diatopic Schiff bases, and it was shown that they bind to Zn^2+^. According to DFT calculations, this binding results in six possible trinuclear circular diastereomers, including four helicate ones.

An interesting π–metal interaction between triptycene and a mononuclear Ag(I)-containing macrocycle was identified in a host–guest system (Figure 4b) [161]. More precisely, the NMR titration experiments revealed that two mononuclear Ag(I) from guests interact with one triptycene molecule. The molecular modeling reveals that each atom interacts with different aromatic rings of the triptycene; however, this scenario can be realized via two different mutual configurations of two Ag-*syn* and *anti*.

**Figure 5:**
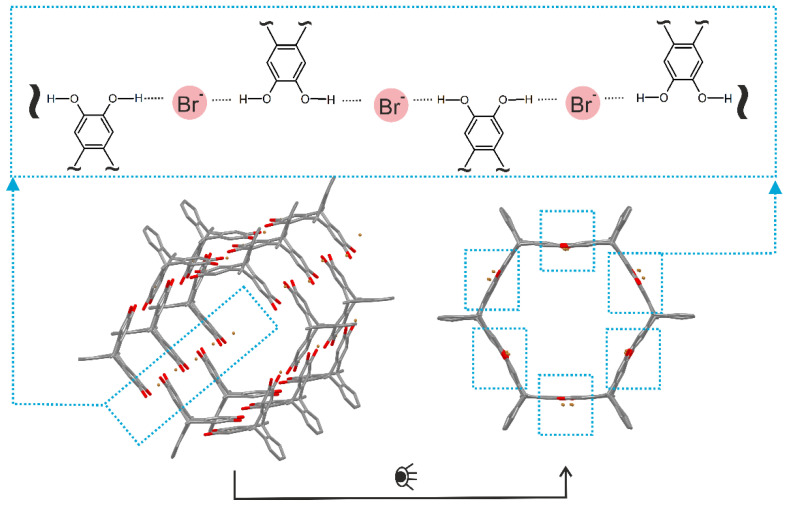
The tetrahydroxytriptycene molecule and a three-dimensional nanotube architecture, which is possible due to the weak O–H∙∙∙Br^−^ hydrogen bonds [162].

Various triptycene derivatives form crystal structures with interesting properties. For example, a hexa- and tetrahydroxytriptycene molecules can form a three-dimensional nanotube architecture when the crystallization is conducted in the presence of bromide anion (Figure 5) [162].

Weak O–H∙∙∙Br^−^ hydrogen bonds are mainly responsible for this unique nanotube scaffold. Interestingly, the structure with tetrahydroxytriptycene is stable in vacuum, does not decompose after 24 h of heating at 105 °C, and can be stored for a few days in water.

**Figure 6:**
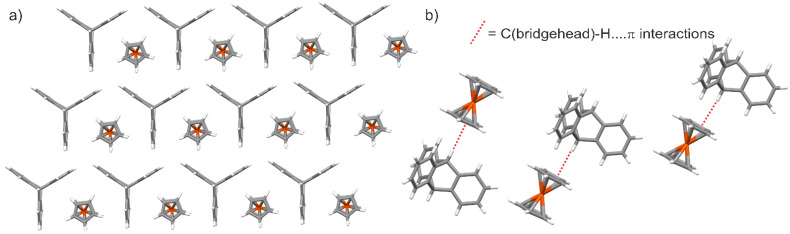
The triptycene–ferrocene cocrystal: (**a**) a honeycomb fragment showing the efficient packing within the cocrystal; (**b**) the interaction between the C(bridgehead)-H and the aromatic ring of ferrocene [163].

Singh investigated the interaction between triptycene and various ferrocenes [163]. They can form a cocrystal with a honeycomb pattern (Figure 6a). The interaction between the C(bridgehead)-H and the aromatic ring of ferrocene was observed in the triptycene–ferrocene cocrystals (Figure 6b). The C–H···π interaction was also found present in cocrystals of triptycene with different ferrocene derivatives. Interestingly, in contrast to the crystal of pure triptycene, in some triptycene cocrystals, a π–π interaction between triptycene units was observed.

### 3.2. Photo-Physical Properties of Some Triptycenes

**Figure 7:**
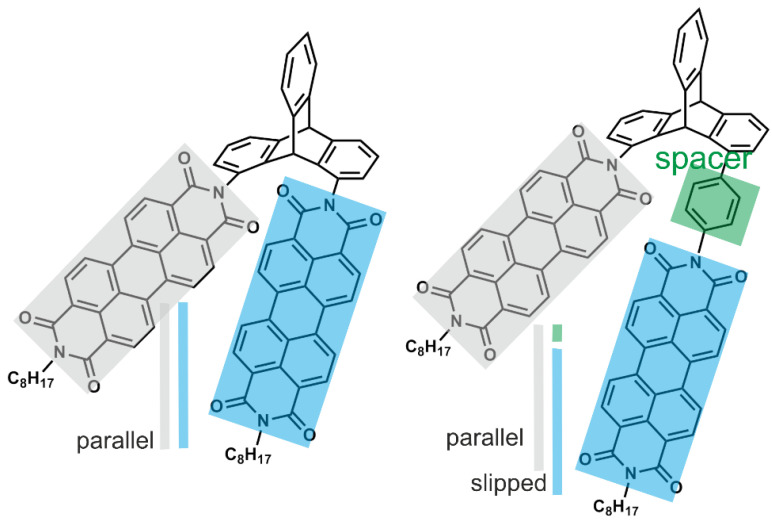
Triptycene utilized as a spacer between two π–π stacking chromophores [164].

The triptycene skeleton was also employed as a convenient spacer for the investigation of excitation energy transfer in two exemplary π–π stacking chromophores (Figure 7) [164].

In the first model, two of the same chromophores were mounted on positions 1 and 8 of the triptycene skeleton in a way that these two chromophores were piled on top of one another. In the second model, both chromophores were located on the same positions; however, one of the chromophores was separated from the triptycene unit by the inserted phenyl ring. As a result, the two chromophores are parallel to each other, but they are slipped relative to each other. The introduction of the phenyl spacer decreases the electronic coupling, which finally leads to the less stable excimer state and higher near-infrared (NIR) excimer absorption energy. Therefore, slipped-stacked structures have a lower tendency to form excitation traps.

**Figure 8:**
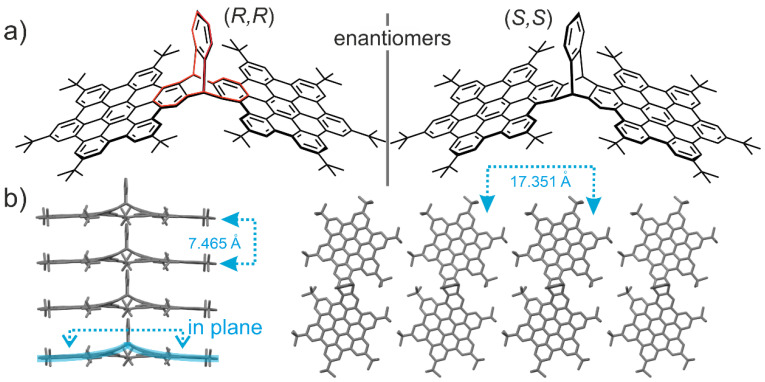
(**a**) The (*R*,*R*) and (*S*,*S*) enantiomers of the exaphenylbenzene triptycene derivative; (**b**) molecular arrangement of the (*S*,*S*) enantiomer in the crystal [138].

The triptycene molecular framework is employed as a building block for cumulene molecular scaffolds, which are attractive for materials science because of their optical activity. Wada presented the synthesis of a cumulated aromatic system with triptycene as a core [138]. The triptycene molecular scaffold was extended by two hexabenzocoronene units. The presented approach involved *rac*-2,6-diiodotriptycene, which was coupled with 4-*tert*-butylphenylacetylene in the Sonogashira–Hagihara cross-coupling reaction. The obtained product was subsequently converted to *rac*-2,6-disubstituted triptycene with two hexaphenylbenzene units. Before the next step, the racemic mixture was separated into two enantiomers by chiral HPLC. The critical step in the presented protocol involved the aromatization of the hexaphenylbenzene derivatives by oxidative dehydrogenation with FeCl_3,_ which yields two enantiomers (Figure 8a). At room temperature and in the dichloromethane/hexane solution, the racemic mixture displays a tendency towards conglomerate crystallization. As a result, a single crystal with only one (*S*,*S*) enantiomer was isolated and X-ray analysis revealed that the molecules are packed one above the other in a way that they create a column (Figure 8b). The distance between consecutive molecules within the column is 7.656 Å, while between neighboring columns the distance is 17.351 Å (Figure 8b). The two polyaromatic rings of a single triptycene unit are almost in-plane, probably because of their homoconjugation. Interestingly, molecular dynamics simulation showed that the two wings are wobbling in a way that the average structure of these wings becomes wrapped, which introduces an extra chiral structural parameter influencing the CD optical activities of both enantiomers. Regarding the optical properties, the luminescence experiments revealed that the enantiomers can emit right- and left-handed circularly polarized light on account of their chirally bent structure.

In the last few years, some papers concerned with the properties of unsubstituted triptycene were presented. For example, it turns out that the parent triptycene can be utilized for growing polyhedral crystals, which are of much interest owing to their potential applications in electronics and optics [165]. The presented research revealed that triptycene can form nanosheets and polyhedra of quasi-decahedra. Extensive investigation carried out by various electron microscopy methods (TEM, SAED, FESEM) and X-ray crystallography indicated that the growth of polyhedral crystals can be controlled with appropriate surfactants such as CTAB and P123.

It also turned out that triptycene tribenzoquinone can also form a crystal with a band structure, with Dirac cones and flat band profiles [166]. Such crystals with an exotic band can be obtained by electrochemical reduction of a TT/RbClO_4_ mixture. This yields high-symmetry hexagonal crystals belonging to the *P*6/*m* space group of Rb_3_TT·2H_2_O, in which the TT molecules form a honeycomb structure through π-package-type interactions. Interestingly, a similar honeycomb structure was observed for the Li_3_TT·2H_2_O crystals; however, these crystals belong to the triclinic *P*1 space group, and therefore their symmetry is low, leading to the loss of Dirac cones and flat bands.

Mizoguchi presented a theoretical interplay between the structure of triptycene derivatives and the electronic properties of polymerized triptycenes by employing a molecular orbital approach for the identification of driving forces behind the flat band topology and Dirac cones [167].

## 4. Conclusions and Outlook

Since the discovery of triptycenes, great progress has been made regarding their synthetic methodology and the understanding of inter- and intramolecular interactions that involve triptycenes. Several new synthetic approaches have been developed in the last few years, and progress has been made in the context of sterically congested triptycenes and regioselective synthesis of various derivatives. This review also demonstrates the potential of triptycenes in catalysis, where these unique frameworks are employed as ligands with various steric and chiral properties, particularly in palladium-based cross-coupling approaches. The progress in triptycene synthesis has produced structures with interesting properties. Therefore, in parallel with the development of the synthetic methodology, efforts have been made to understand the physico-chemical properties of triptycene derivatives. Intramolecular interactions between substituents within the triptycene unit were investigated, and new types of bonding and nonbonding interactions were detected. These interactions influence intramolecular interactions between triptycene molecules and between triptycenes and other molecules or surfaces. This is of crucial importance for the rational design of functional materials. Additionally, many triptycenes have very interesting optical properties, which makes them attractive for optoelectronic applications. In particular, the aromatic rings system present in triptycenes can be π-extended with a desired optical activity in mind. Finally, the properties of triptycene molecules affect the crystals formed from them, which very often results in useful properties of the crystal, such as interesting band structure with the Dirac cones and flat band profiles.

In conclusion, the advances in synthetic methodology and a better understanding of the relation between triptycene structures and their properties have been very significant. Moreover, the progress of triptycene synthesis and comprehensive insight into the nature of these interactions is of central concern for the rational design of triptycene-based functional materials. However, some questions remain open, and the chemistry of triptycenes and their applications will still be developing.

## Figures and Tables

**Table 1:** Selected experimental ^1^*J*(Si,C_Ph_) and ^1^*J*(H,Si) spin–spin coupling constants (Hz) within the -SiH_2_(Ph) group of two rotamers (see Figure 2) of TRPCl.

	TRPCl-R1	TRPCl-R2
^1^*J*(Si,C_Ph_)	68	-
^1^*J*(Si,C_Ph_)	-	77
^1^*J*(H1,Si)	214	215
^1^*J*(H2,Si)	214	190

## Data Availability

Not applicable.
